# Patient Navigation—Who Needs What? Awareness of Patient Navigators and Ranking of Their Tasks in the General Population in Germany

**DOI:** 10.3390/ijerph19052846

**Published:** 2022-03-01

**Authors:** Susanne Schnitzer, Raphael Kohl, Hella Fügemann, Kathrin Gödde, Judith Stumm, Fabian Engelmann, Ulrike Grittner, Nina Rieckmann

**Affiliations:** 1 Charité—Universitätsmedizin Berlin, Corporate Member of Freie Universität Berlin and Humboldt Universität zu Berlin, Institute of Medical Sociology and Rehabilitation Science, Charitéplatz 1, 10117 Berlin, Germany; raphael.kohl@charite.de; 2 Brandenburg Medical School Theodor Fontane, Institute of Social Medicine and Epidemiology, 14770 Brandenburg an der Havel, Germany; hella.fuegemann@mhb-fontane.de; 3 Charité—Universitätsmedizin Berlin, Corporate Member of Freie Universität Berlin and Humboldt Universität zu Berlin, Institute of Public Health, Charitéplatz 1, 10117 Berlin, Germany; kathrin.goedde@charite.de (K.G.); nina.rieckmann@charite.de (N.R.); 4 Charité—Universitätsmedizin Berlin, Corporate Member of Freie Universität Berlin and Humboldt Universität zu Berlin, Institute of General Practice and Family Medicine, Charitéplatz 1, 10117 Berlin, Germany; judith.stumm@charite.de; 5 Kassenärztliche Bundesvereinigung (KBV), Geschäftsbereich Sicherstellung und Versorgungsstruktur, Abteilung Versorgungsstruktur, 10592 Berlin, Germany; fengelmann@kbv.de; 6 Charité—Universitätsmedizin Berlin, Corporate Member of Freie Universität Berlin and Humboldt Universität zu Berlin, Institute for Biometry and Clinical Epidemiology, Charitéplatz 1, 10117 Berlin, Germany; ulrike.grittner@charite.de; 7 Berlin Institute of Health at Charité—Universitätsmedizin Berlin, Charitéplatz 1, 10117 Berlin, Germany

**Keywords:** patient navigation, health information, emotional support, social support, population survey, sociodemographic characteristics, chronic disease, subjective health

## Abstract

The aim of the present study was to investigate the awareness of patient navigation (PN) in the general population in Germany and to assess which navigator tasks are considered most important. The analysis drew on a 2019 nationwide telephone survey of 6110 adults. We compared rankings of emotional support, administrative support and information among respondents with and without experience of patient navigation. One-fifth of the sample reported having heard of PNs; 13% of this group already had experience with PN. In both groups, the majority (>47%) considered assistance with applications to be most important. This was particularly the case among younger adults and those with a chronic disease. Within the inexperienced group, higher educated people had higher odds of ranking provision of information as most important for them, whereas women and those without a partner had higher odds of ranking emotional support as the most important task. This study shows that the majority of people predominantly expect PN services to offer administrative support, irrespective of their socioeconomic and health status. Whether these expectations are met by the diverse existing PN programs, which often have a strong focus on other tasks (e.g., increasing health literacy), has yet to be evaluated.

## 1. Introduction

A chronic disease raises a variety of questions for those affected. These may concern acute medical treatment, rehabilitation and other therapy. Furthermore, questions about social security, social benefits and assistance with everyday living/homecare may arise. In outpatient care, the degree of assistance that patients receive in managing and coordinating their own healthcare varies greatly between but also within healthcare systems. In many cases, patients are left unassisted in the coordination of all medical, psychological, social and legal aspects related to their disease management. The presence of multiple chronic conditions, absence of an informal support system and low health literacy may further complicate this. These barriers also exist in the German healthcare system. Above all, research has revealed that there is little coordination of patients’ continued care in Germany [1], difficulties due to complex care in multimorbid patients [2], fragmentation of the German healthcare system and separate organization of the inpatient and outpatient sector [3,4]. In addition to these system barriers, on the patients’ side, low health literacy [5]—especially of vulnerable groups, such as migrants or lower educated people [6,7]—or limited knowledge about further support offers [8,9,10] can restrict access to optimal healthcare.

In order to support patients over a longer period of time, patient navigation (PN) models have been deployed internationally and, more recently, in Germany [11,12,13,14,15,16]. Patient navigators (PNs) guide and support patients in gaining access to timely care and handling the increasingly complex healthcare system or treatment regimes [12,14,17]. They aim to support patients to organize their healthcare according to their individual needs and to optimize their care trajectory [11,14]. Patient navigators provide practical support (e.g., with applications, organization of appointments), give advice on social care issues and draw attention to existing support offers. In short, patient navigators (PNs) assist, inform, advise and guide patients through the healthcare system. They help patients to find their way through the healthcare and, if needed, adjacent care systems and support them in finding a competent institution or contact person for their concerns, needs and problems. Patient navigation ideally connects all healthcare sectors for patients, i.e., both in the hospital and at the doctor’s office or in rehabilitation [18]. There is only a minimal consensus on the tasks and functions of PNs [19,20]. On the basis of qualitative interviews and focus group discussions with nurses, specialists and family doctors, Fillion and colleagues proposed a bi-dimensional framework that encompasses certain core tasks of navigators. The first dimension refers to the continuity of care, and the second dimension relates to patient and family empowerment [19]. Continuity of care was further divided into informational continuity (use of information, disease- or person-focused) and management continuity (e.g., matching unmet needs with services). The second dimension, empowerment, includes concepts such as supportive care, e.g., addressing patients’ emotional and psychological needs [19].

In Germany, various—mainly indication-specific—patient navigation models have been developed in recent years and are currently being tested for their feasibility and efficiency [21,22]. These are model projects and patient navigation is currently not a widely implemented reimbursable service in statutory health insurance (in Germany, insurance is mandatory and about 90% of the population is covered by statutory health insurance). The majority of these patient navigation programs have been developed using an expert-driven approach and are based on the concept of ‘ideal’ patient pathways and evidence-based patient care for specific diseases. In this context, the roles and tasks of the navigators are defined a priori. However, to our knowledge, patients’ perspectives on which tasks of patient navigation are most important have not been investigated systematically. In particular, this raises the question of the specific needs of different population groups or, in other words, who needs what most? The subjective needs of patients are an important requirement for the successful implementation of patient navigation models in healthcare practice. As there is little research on this topic, our study sheds some light on it.

### Aim of the Study

The aim of the present study was to investigate the awareness of PN in the general population and to assess which navigator tasks are considered most important in several population subgroups according to sociodemographic and health status characteristics and previous experience with PN.

## 2. Materials and Methods

### 2.1. Study Design

Since 2008, an expert group of the German National Association of Statutory Health Insurance Physicians (Kassenärztliche Bundesvereinigung, KBV), in cooperation with the Institute of Medical Sociology and Rehabilitation Science of Charité University and the research institute Forschungsgruppe Wahlen (FGW), has conducted an annual representative population survey among all German-speaking adults living in households with a landline phone on various topics in outpatient healthcare. The analyses presented here are based upon the 2019 survey. A random sample was generated through regional stratification of the population, selection of landline phone numbers via randomized last digit dialing and selection of the respondent through the last birthday method. Computer-assisted telephone interviews (CATI) were conducted in the German language by the FGW between 11 March and 29 April 2019. The data were weighted for the number of landlines and persons per household, as well as for gender, age and education according to their nominal distribution across the adult population in Germany [23]. The weighted sample is representative for the German-speaking adult population and comprises 6110 persons (Table 1).

### 2.2. Assessment of PN Awareness and Importance of Navigator Tasks

Within an expert group of the NAVICARE research network (authors S.S., H.F., K.G., J.S.), the assessment of PN awareness and the most important tasks of PNs from the population’s perspective were discussed. Finally, following the theoretical framework proposed by Fillion and colleagues [19], described above, and taking into account the results from a qualitative interview study with twenty lung cancer and twenty stroke patients [1], three main tasks of PNs were defined. Respondents were asked to select which one they consider the most important task. First, participants were asked whether they had heard of PNs before:

‘In healthcare, there is a service provided by PNs who support and advise patients over a longer period of time after an acute illness/event such as stroke, or a longer-lasting disease such as cancer, e.g., in filling out applications. Have you heard of PNs?’

Subsequently, a filter was set: All those who answered ‘yes’ here were asked to indicate whether they had already had experience with a PN. This population constitutes the ‘experienced’ subsample in the present study. Respondents who had already heard of PNs but had not yet had any experience with PNs themselves constitute the ‘inexperienced’ subsample. Both subsamples were asked about their views on the most important task of PNs as follows: ‘Which of the following three tasks of PNs would be/was most important to you?

- assistance with administrative matters, e.g., applications for rehabilitation care,
- provision of healthcare information,
- counseling and support for emotional problems resulting from the disease’.

Multiple answers were not possible, i.e., respondents had to choose one task.

### 2.3. Sociodemographic Characteristics and Health Status

PN awareness and the support needs/perception of the most important tasks may vary according to socioeconomic and health status. The following characteristics and subgroups were analyzed: Age (18–64/≥65 years), gender (female/male), educational attainment (high school/no high school), current partnership (no/yes), region of current residence (East/West Germany), residential area (rural ≤ 5000; small town 5001–100,000; urban > 100,000), chronic illness (yes/no) and subjective health status (excellent, very good, good/less well, bad).

### 2.4. Statistical Analysis

For both subsamples (experienced/inexperienced), three dichotomous variables on the most important tasks (support with applications, provision of information, emotional support) were generated (0 = not most important/1 = most important). Associations with sociodemographic and health characteristics were analyzed using multiple binary logistic models. The analyses are considered exploratory. Altogether, we explored six models: M1a. application, M2a. information, M3a. emotional support (dependent variables of the inexperienced) and M1b. application, M2b. information, M3b. emotional support (dependent variables of the experienced). For each task, odds ratios (OR) and 95% confidence intervals (CI) were calculated. The two-sided level of significance was set at 5%. No adjustment for multiple testing was applied.

The number of refusers on the key variables were negligible, with 0.03% for knowledge of (‘have you heard of PNs?’) and 0.15% for experience with PNs (‘have you already had experience with a PN?’). Respondents who refused to answer these questions were excluded from further analysis. In the experienced group, the rate of missing data on the question about the most important task of PNs was 9%; in the inexperienced group, it was 12%. Refusers on the questions on the most important tasks were included and coded as 0 (not most important) for each variable. Randomness of missing data was analyzed using the Chi² test with sociodemographic and health characteristics. Missing data occurred more often in older participants among the experienced (*p* = 0.02) and inexperienced group (*p* < 0.001) and in those with lower education among the inexperienced group (*p* < 0.001). All statistical analyses were performed using SPSS version 27.0 [24].

## 3. Results

About one-fifth of respondents (*n* = 1275/20.9%) had already heard of PNs. Of these, the majority had no prior experience with PN (inexperienced, *n* = 1105/86.8%), while a small group had prior experience (experienced, *n* = 168/13.2%).

### 3.1. The Most Important Tasks of PNs

In the inexperienced group, most people ranked support with applications (*n* = 525/47.5%) as the most important task of a navigator, followed by emotional support (*n* = 282/25.6%) and provision of information (*n* = 159/14.3%) (other support = 12.6%). In the experienced group, this ranking was slightly different. Support with applications was also ranked highest by about half of this subsample (*n* = 87/51.7%), followed by provision of information (*n* = 40/23.7%); few of the experienced ranked emotional support (*n* = 25/15.2%) as the most important task (other support = 9.4%).

### 3.2. Support with Applications

**Figure 1:**
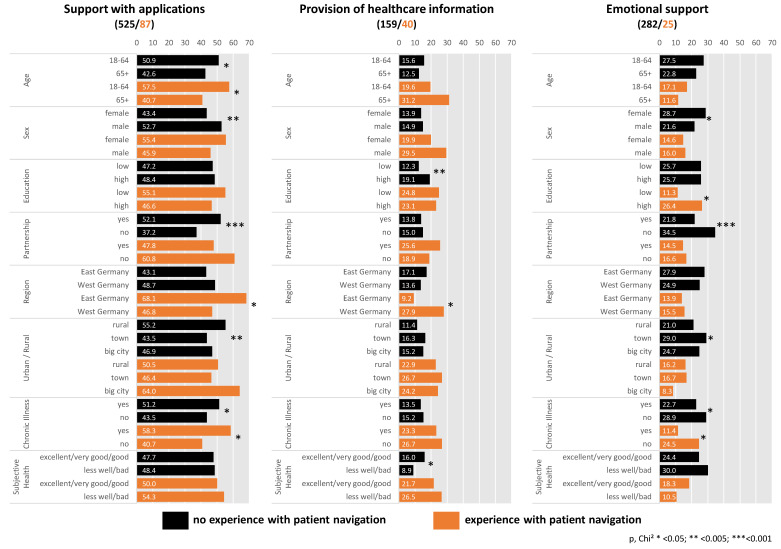
Differences (%, *p*) between the most important tasks and sociodemographic characteristics within both groups (persons with and without experience of patient navigation).

The descriptive results in Figure 1 show that within the inexperienced group, persons living in a partnership, younger respondents, men, chronically ill persons and respondents from rural regions assessed support with applications as the most important task of PNs (Figure 1). Younger respondents and chronically ill persons in the experienced group also answered more often than older respondents and persons without a chronic disease that support with applications is most important for them.

These results were confirmed by the results of the multiple regression models, with one exception: Within the inexperienced group, there was no substantial effect with city size after adjusting for the other characteristics. However, within the experienced group such an effect appeared: fewer people in rural areas (OR: 0.29; CI: 0.10, 0.89) and small towns (OR: 0.36; CI: 0.13, 0.98) than people in big cities assessed support with applications as most important (Table 2).

### 3.3. Provision of Healthcare Information

Within the inexperienced group, more highly educated than less educated persons and more persons with very good health than with poor health named the provision of health information as the most important task of PNs (Figure 1). In the experienced group, people from West Germany in particular assessed this task as the most important (Figure 1). The multiple regression analysis showed the same results. Within the inexperienced group, higher educated people had higher odds than lower educated people (OR: 1.55; CI: 1.06, 2.27) and people with bad health had lower odds than people with good health (OR: 0.54; CI: 0.32, 0.89) of ranking information as most important for them. Within the experienced group, more West than East Germans (OR: 3.59; CI: 1.06, 12.18) ranked information as the most important task (Table 2).

### 3.4. Emotional Support

For the inexperienced without a partner, emotional support from PN was identified as most important more often than for those with a partner. More women than men and more persons without a chronic disease compared with chronically ill persons rated emotional support as the most important task. The latter also applies to the experienced—more respondents without a chronic disease than with a chronic disease identified emotional support as the most important role of a PN. In the experienced group, the disproportionately high percentage of highly educated persons who stated that emotional support was most important is a striking result (Figure 1). Again, the results remained consistent in the multiple regression analyses. Within the inexperienced group, women, those without a partner and those without a chronic disease had higher odds of ranking emotional support as the most important task, whereas among the experienced, higher education was associated with emotional support as the most important task of a PN (Table 2).

## 4. Discussion

The present study provides evidence on the general population’s views on PN tasks. Next to assessing the level of awareness of PNs in Germany, the objective of the study was to ascertain the most important tasks of PNs from the viewpoint of different population groups. ‘Who needs what most?’ was the guiding research question.

### 4.1. Comparison between Persons with and without Experience of PNs

Overall, about one-fifth of the population in Germany reported having heard of PNs, with 13% of this group already having had experience with this still fairly new care model. Assuming that prior experience with PNs has an influence on how the various tasks of navigators are prioritized by the patients, analyses were conducted separately for respondents with and without experience. The results show that there are both similarities and differences between the two groups. For both groups, it appears that the majority of respondents consider assistance with applications to be most important. For the inexperienced, however, emotional support is then cited second most often, while for the experienced, the second-ranked task was providing information.

There is wide evidence on the high relevance of health information as part of health literacy, which in turn influences people’s health [7,25]. Broad activities have been carried out both nationally and internationally to disseminate health information to specific target groups or the general population—most recently during the COVID-19 pandemic [26,27,28]. However, the results of the present work indicate that in the case of a chronic illness or disease event, practical assistance in bureaucratic matters is even more important for patients than receiving information about their disease. Even though our results refer to Germany with its specific application system, e.g., in the field of rehabilitation care, studies from other countries also confirm the high relevance of bureaucratic support for patients in the healthcare system. A qualitative Canadian study explored caregivers’ experiences caring for a child or youth with complex care needs, and their experiences and satisfaction as clients of a patient navigation center. As participants reported overwhelming organizational tasks, employed navigators supported the caregivers in bureaucratic matters and thus, among other things, improved the quality of life of the caring parents [29]. A study from the USA explored oncology navigators’ perceptions of cancer-related financial burden and financial assistance resources via an online survey. Seventy-eight respondents participated in the survey, reporting that commonly identified barriers for patients obtaining assistance included lack of resources, lack of knowledge about resources and complex/duplicate paperwork [30]. This is in line with our study in which those with experience rated support with applications and the provision of information as most important for them more often than emotional support. Of course, this does not mean that emotional support is unimportant for this group of patients. One explanation for the relative lower ranking could be that this support need is currently better covered by existing support networks for chronically ill people and their psychological burdens. Psycho-oncology can be cited as a prominent example here [31]. In light of the current COVID-19 pandemic situation, the question which arises is whether respondents’ prioritizing of PNs’ tasks may change due to COVID’s direct and indirect effects. In particular, it is conceivable that emotional support might be given a higher priority as people are more isolated and have more difficulties accessing support systems and healthcare. Simultaneously, it is possible that due to the increased barriers to gaining timely and adequate access to healthcare, people would rank practical/bureaucratic support as the most important tasks of PNs. Studies conducted during a future pandemic may use our binary coding system for a prompt assessment of the most important tasks of PNs; in such cases, the results should be analyzed depending on additionally integrated questions on the specific pandemic situation.

### 4.2. Support with Applications

In both groups there were associations with age and a chronic disease, i.e., regardless of experience with PN, support with applications was most important for younger respondents and respondents with a chronic disease. Thus, the priority here was not the need for health information, although a study showed that younger people in particular know little about health issues [32]. Due to their experiences, chronically ill patients, in turn, have a fairly good knowledge of health issues, so that their higher need for practical support seems plausible. This interpretation is in line with the results of a nationwide survey with chronically ill patients in the Netherlands [33]. In the experienced group, it is furthermore noteworthy that a high ranking for application support is particularly likely in big cities. Whether this is rooted in differences in individual support systems or an expanded understanding of healthcare providers’ roles in rural versus urban areas in Germany has yet to be investigated.

### 4.3. Provision of Health Information

In the inexperienced group, the provision of health information by a PN is prioritized particularly often by well-educated respondents and respondents with good health. It is important to point out that a higher need for information does not necessarily mean a higher information deficit. On the contrary, with regard to educational attainment, various studies revealed a significant correlation between a high level of education and a high level of information, as well as a higher level of health literacy that goes along with it [32,33]. Health literacy includes the ability and desire to gather, obtain and understand health information [5,34,35]. In this respect, the results point to higher proactive behavior of higher educated people. This was confirmed by a study on informal caregivers based on a representative population survey in Germany. The results revealed that the odds of better educated people talking with their general practitioner about their burden of informal caregiving were significantly higher than for those with a lower education level [34]. These findings shed light on the needs of the population with low education levels, as they indicate lower proactive support seeking within this group. Studies in Germany showed that people with basic education are more likely to have difficulties in understanding health information than people with higher education [7,35]. This could be one reason for their lower proactive behavior compared to the higher educated. A study by Tille and colleagues in Germany revealed that health information seems to be insufficiently tailored to individuals aged 50 years and above as well as to those with intermediate and basic education [7]. Consequently, and in line with these results, it can be assumed that tailoring health information and materials to the competences of those with lower education may further facilitate these groups’ understanding of health issues and foster patient empowerment.

The higher odds of people in West Germany than people in East Germany ranking the provision of information as a key task of PN is a noteworthy result in the experienced group. According to the results discussed above, this does not necessarily mean that there is a lower level of knowledge in West Germany. Also, it is possible that cultural differences due to previous exposure to differing healthcare systems in East and West Germany may account for this.

### 4.4. Emotional Support

The evaluation of emotional support was influenced by different characteristics—depending on already having had experience with PN or not. In the inexperienced group, women were more likely than men to prioritize emotional support. This result is confirmed by study results that show that women provide and receive more emotional support [36], which in turn can be explained by traditional role concepts and social norms [37,38,39]. However, it is notable that this association is no longer present in the experienced group. Here, the question that arises is whether the provision of a PN might have covered the emotional needs of women. The patient navigation studies currently being undertaken in Germany may well provide important insights here (for an overview of current patient navigation programs in Germany see [22]).

The protective effect of a partnership and social networks on health is confirmed by a number of studies [40,41,42,43,44]. Thus, the result is plausible that respondents without PN experience and without a partner were more likely than respondents with a partner to prioritize the emotional support of a PN. Analogous to the gender result discussed above, here too, the question is whether the support provided by a PN could have partly contributed to the result that respondents without a partner but with PN experience did not rank this form of support as a key task more often than respondents with a partner.

The result within the experienced group that better educated people rated emotional support as the most important task more often than less educated people points to a need for further research, as the background to this is unknown. A study by Oedekoven et al. on physical and mental burdens of informal caregivers showed that higher educated people were affected more often than lower educated people by mental burdens due to their caregiving situation [45]. According to these results, it seems that prevention measures and patient navigation programs should be tailored more precisely to the educational background of patients in order to meet their specific needs more effectively.

### 4.5. Strengths and Limitations

This study has substantial strengths, including a cohort of a large nationwide representative sample as well as the first ever assessment of patient navigation awareness and the population’s view on PNs’ most important tasks. Utilizing this sample, we provide evidence of the prioritized needs of different population groups regarding patient navigation. As a limitation of the study relating to the experienced group, the experience with navigation may be somewhat heterogeneous as patient navigation is not a care model that is regularly available to patients in Germany yet. On the one hand, the group may be comprised of participants from various model projects that are currently being evaluated in Germany. On the other hand, they may have experienced support from providers such as community care points or advice centers run by the Public Health Departments, which do not entirely fulfill the definition of patient navigation provided at the start of this paper but offer some forms of care management. Despite these differences, the study results indicate which support services patients seek most urgently.

Furthermore, the data were collected by telephone, and only people with a landline were contacted [7,23]. This might have resulted in a higher proportion of older persons being reached [46]. Older people refused to answer the question about the most important tasks of PNs more often than younger people (in both groups) and lower educated people more often than higher educated people (in the inexperienced group). Thus, differences in age and education may have been affected by differential refusal. However, as rates of missing data are low, we assume these effects are minor.

## 5. Conclusions 

This study shows that the majority of people predominantly expect PN services to offer administrative support. However, there were variations in expectations by educational level, age, region and city size, health status, gender, marital status and prior experience with a patient navigation program. If no prior experience with PN programs exist, younger respondents and chronically ill people in particular prioritize support with applications as the most important task, while women and people without a partner rank emotional support and well-educated people the provision of health information as the most important task of a PN. If experience has already been gained with a PN, people in big cities rank support with applications most often as the most important task, while the provision of health information is prioritized more often by West than by East Germans and emotional support more often by better than by lower educated people.

In sum, results indicate that the social determinants of health, such as educational background, marital status or gender, should be recognized for tailored patient navigation models in order to provide better care and support to those in need. For successful broad-scale implementation of PN programs, it seems advisable to design the models flexibly so that the focus of the programs and navigators’ tasks can vary depending on different population groups and their prioritized needs.

## Figures and Tables

**Table 1:** Sociodemographic baseline data (weighted sample).

		*n* = 6110 *	% [95% CI]
**Age**	18–64	4294	70.3 [69.1; 71.4]
	65+	1815	29.7 [28.6; 30.9]
**Sex**	female	3196	52.3 [51.1; 53.6]
	male	2914	47.7 [46.4; 48.9]
**Education**	low	4122	68.3 [67.1; 69.4]
	high	1916	31.7 [30.6; 32.9]
**Partnership**	yes	4341	71.4 [70.2; 72.5]
	no	1741	28.6 [27.5; 29.8]
**Region**	East Germany	1063	17.4 [16.5; 18.4]
	West Germany	5047	82.6 [81.6; 83.5]
**Urban/Rural**	rural	1781	31.2 [30.0; 32.4]
	town	2603	45.6 [44.3; 46.9]
	big city	1326	23.2 [22.1; 24.3]
**Chronic Illness**	yes	2885	47.6 [46.3; 48.8]
	no	3177	52.4 [51.2; 53.7]
**Subjective health**	excellent/very good/good	4716	77.9 [76.8; 78.9]
	less well/bad	1342	22.1 [21.1; 23.2]

* Difference to 6110: missing data.

**Table 2:** Associations between the most important tasks within both groups and sociodemographic characteristics (multiple binary logistic regressions, OR 95% CI).

	No Experience with Patient Navigation			Experience with Patient Navigation		
	M1a. Application	M2a. Information	M3a. Emotional	M1b. Application	M2b. Information	M3b. Emotional
	OR [95% CI]	OR [95% CI]	OR [95% CI]	OR [95% CI]	OR [95% CI]	OR [95% CI]
Age						
18–64	*1.33 [1.02, 1.74]*	1.15 [0.78, 1.69]	1.33 [0.97, 1.81]	*4.68 [1.86, 11.80]*	0.56 [0.22, 1.40]	0.64 [0.19, 2.17]
65+	1	1	1	1	1	1
Sex						
female	1	1	1	1	1	1
male	1.55 [1.20, 2.00]	0.98 [0.69, 1.40]	*0.66 [0.49, 0.89]*	0.48 [0.21, 1.07]	1.55 [0.68, 3.51]	1.27 [0.47, 3.45]
Education						
low	1	1	1	1	1	1
high	1.01 [0.76, 1.35]	*1.55 [1.06, 2.27]*	0.92 [0.66, 1.27]	0.56 [0.22, 1.45]	0.73 [0.27, 1.99]	3.31 [1.05, 10.41]
Partnership						
yes	*1.75 [1.32, 2.32]*	0.82 [0.56, 1.21]	*0.53 [0.39, 0.71]*	0.53 [0.23, 1.24]	1.66 [0.68, 4.07]	0.98 [0.33, 2.97]
no	1	1	1	1	1	1
Region						
West Germany	1.40 [1.02, 1.92]	0.70 [0.46, 1.06]	0.75 [0.53, 1.07]	0.38 [0.14, 1.01]	3.59 [1.06, 12.18]	0.90 [0.27, 2.97]
East Germany	1	1	1	1	1	1
Urban/rural						
rural ≤ 5000	1.33 [0.94, 1.88]	0.85 [0.52, 1.41]	0.84 [0.56, 1.26]	*0.29 [0.10, 0.89]*	1.03 [0.32, 3.24]	3.15 [0.67, 14.67]
town 5001–100,000	0.81 [0.59, 1.12]	1.14 [0.74, 1.76]	1.27 [0.89, 1.83]	*0.36 [0.13, 0.98]*	1.65 [0.59, 4.65]	1.82 [0.46, 7.25]
big city	1	1	1	1	1	1
Chronic illness						
yes	*1.68 [1.29, 2.20]*	0.91 [0.63, 1.32]	*0.66 [0.49, 0.89]*	*4.99 [2.01, 12.42]*	0.54 [0.21, 1.41]	0.37 [0.12, 1.17]
no	1	1	1	1	1	1
Subjective health						
less well/bad	1.00 [0.74, 1.37]	0.54 [0.32, 0.89]	1.30 [0.92, 1.84]	0.60 [0.26, 1.39]	1.46 [0.60, 3.53]	0.89 [0.28, 2.90]
excellent/very good/good	1	1	1	1	1	1
R² (Nagelkerke)	0.073	0.037	0.061	0.047	0.122	0.148

OR = odds ratios; 95% CI = and 95% confidence intervals, significant values at the 5% level are highlighted in italics.

## Data Availability

The datasets generated and analyzed during the current study will be stored in a non-publicly accessible repository. The access information is available from the corresponding author on reasonable request.
